# Casting process optimization of stainless-steel pump impellers using finite element simulation

**DOI:** 10.1038/s41598-026-62136-8

**Published:** 2026-07-17

**Authors:** Mahmoud A. Essam, Noha M. Abdeltawab, Ahmed Y. Shash, Mostafa M. El-Sayed

**Affiliations:** 1 Mechanical Engineering Department, Higher Technological Institute (HTI), 10th of Ramadan City, Egypt; 2 Faculty of Engineering, City University of Cairo (CUC), New Heliopolis City, Egypt; 3 Faculty of Engineering and Materials Science, German University in Cairo, Cairo, Egypt; 4 Mechanical Design and Production Engineering Department, Faculty of Engineering, Cairo University, Giza, 12316 Egypt; 5 Manufacturing and Production Engineering Department, Modern Academy for Engineering and Technology, Cairo, Egypt

**Keywords:** Centrifugal pump impeller, Casting simulation, Shrinkage porosity, Directional solidification, Gating and feeding optimization, Engineering, Materials science

## Abstract

This study employed ProCAST numerical simulation to assess and enhance the casting design of a stainless-steel CF8M pump impeller by analyzing six gating and feeding configurations. The simulations were executed at a pouring temperature of 1650 °C, an initial mould temperature of 25 °C, and an estimated filling duration of 20 s. The examined configurations comprised a baseline horizontal design and modified designs featuring enhanced riser and gate arrangements, mould tilt angles ranging from 5° to 20°, and supplementary ventilation in the final design. The findings indicated that the original design resulted in detrimental temperature distribution and localized final solidification within the impeller body, especially around the hub and blade-hub connections, hence heightening the propensity for shrinkage porosity. The progressive alteration of the riser, sprue, gate configuration, mould angle, and ventilation system optimized the temperature gradient, augmented feeding efficiency, and relocated the final solidification zone towards the riser/feeder. Design 6, optimized with a 20° mould tilt and ventilators, had the most advantageous solidification characteristics and reduced anticipated shrinkage porosity within the functioning impeller body. The findings indicate that numerical simulation can significantly diminish dependence on conventional trial-and-error casting experiments by facilitating virtual evaluation of several design options before to production. This study presents a simulation-based approach for regulating solidification behavior, enhancing feeding efficiency, and reducing shrinkage porosity in the casting of stainless-steel pump impellers.

## Introduction and Literature Review

In a variety of industrial industries, such as water treatment, power generation, chemical processing, oil and gas, and marine applications, centrifugal pump impellers are essential rotating parts that transform mechanical energy into fluid kinetic energy^1^. Impellers must meet strict standards for structural integrity, dimensional precision, and fatigue resistance because of their continuous operation under high rotational speeds, pressure fluctuations, and corrosive environments^2^. Even minute manufacturing flaws might cause premature failure, raise vibration levels, and drastically shorten service life. Therefore, one of the main concerns in foundry engineering is making sure that impellers are produced without defects^2^.

For metallic impellers, casting is still the most popular manufacturing method because it can create intricate geometries at a lower cost than significant machining or additive manufacturing. For stainless steel impellers, sand casting and investment casting are the most often used casting methods. Investment casting is preferable when great dimensional accuracy and thin-walled features are needed, but sand casting is preferred for its flexibility and economic feasibility for making medium-to-large components. However, both procedures are intrinsically vulnerable to flaws brought on by non-uniform cooling, complex molten metal flow behavior, and solidification shrinkage, particularly in parts like impellers that have complex blade geometries and wide variations in wall thickness^2,3^.

According to recent academic and industrial research, casting-related flaws account for between 30 and 70% of rejected components in some production lines, making them a major contributor to foundry trash rates. It has been demonstrated that inadequate gating and feeding design in the case of stainless-steel pump impellers can restrict casting yield to values as low as 41–45%, leading to significant material waste and higher production costs. As a result, increasing casting yield and quality through scientific process design has emerged as a key goal for both industry and research^4^.

Consistently producing impellers without defects is still a difficult undertaking, even with extensive industrial experience in impeller casting. The main challenge is that the casting process is highly linked, with nonlinear interactions between molten metal flow, heat transfer, solidification kinetics, and thermomechanical stresses^3^. The empirical design guidelines and trial-and-error modifications of gating systems, riser diameters, and pouring parameters are still widely used in traditional foundry practice. Even though these methods work well for simple geometries, they frequently fall short for intricate parts like impellers^1^.

Impeller castings that passed visual inspection but had interior shrinkage voids and porosity that were only discovered through machining or numerical analysis are documented in a number of studies. Despite the lack of obvious surface flaws, Jurković et al.‘s investigation of sand-cast AISI 316 L pump impellers revealed shrinkage porosity only after internal machining. Subsequent numerical simulations verified that the likelihood of shrinkage faults in crucial areas above 60%, signifying a basic shortcoming of the conventional inspection and design approach. Furthermore, impeller designs have been pushed toward thinner blades, lower mass, and tighter dimensional tolerances due to growing industrial needs for higher efficiency pumps^2^.

These developments make trial-and-error methods more expensive, time-consuming, and unreliable by further reducing the processing window and increasing sensitivity to heat gradients and feeding circumstances^5^. Among the most frequent quality problems in impeller manufacturing are casting defects such shrinkage porosity, gas porosity, non-metallic inclusions, misruns, and hot tears. Because of their substantial volumetric contraction during solidification and very broad solidification range, shrinkage porosity is especially important in stainless steel impellers. When feeding is inadequate, this defect usually develops in dense areas, blade–hub junctions, and areas that harden last^6^.

Kim et al. investigated the investment casting of centrifugal pump impellers and showed that low casting yield and persistent shrinkage problems were caused by incorrect riser sizing and positioning. Oversized risers and ineffective feeding routes in their original form limited the casting yield to 41.5%. They achieved a far higher yield of 65.2% by utilizing numerical simulation to optimize the runner–riser system, reducing the riser diameter from 133 mm to 66 mm while totally removing shrinkage flaws^7^.

This finding emphasizes the close connection between material waste and defect generation in traditional casting methods. Air entrainment, oxide film formation during mold filling, and turbulent molten metal flow are the main causes of gas porosity and inclusion defects^7^.

Because of the quick variations in cross-sectional area along the blade channels, these flaws are especially troublesome in impeller castings. Kim et al. also demonstrated how irregular flow rates during filling caused gas and inclusion flaws to concentrate on the impeller’s front and rear covers, endangering fatigue resistance and operational dependability. Because trial-and-error approaches don’t provide anything about internal flow patterns, solidification sequences, or defect creation mechanisms, they are essentially insufficient to address these problems^7,8^.

Because each physical trial necessitates mold preparation, melting, pouring, cooling, and inspection, development cycles are lengthy and expenses are expensive. Additionally, empirical changes frequently target symptoms rather than underlying causes, producing less-than-ideal and non-reproducible results. Despite the widespread use of casting simulation in recent years, a rigorous analysis of the literature identifies several unanswered questions. Much of the research that is now available concentrates on the behavior of mold filling or solidification separately, failing to completely integrate flow, thermal, and mechanical phenomena. For example, some studies forecast shrinkage porosity using thermal criteria, ignoring flow-induced turbulence and gas entrapment, which are equally important in impeller castings^9^.

Furthermore, optimization attempts are frequently restricted to a small number of parameters, including gating cross-section or riser size, while ignoring tilt angle, mold orientation, or the relationship between feeding efficiency and flow stability. Although Kim et al. showed that mold tilting significantly reduced gas and inclusion flaws, many impeller-focused studies have not thoroughly examined such solutions. Additionally, many efforts still need quantitative confirmation of simulation results. Fewer studies describe numerical probabilities of defect development or offer systematic comparisons between simulated and experimental yields, even though defect locations are often predicted qualitatively^7^.

This emphasizes the necessity of comprehensive, quantitatively verified frameworks that incorporate finite element analysis, casting simulation, and process optimization, especially designed for intricate impeller geometries. Fundamental physical processes pertaining to fluid flow, heat transfer, phase transition, and stress development control the development of casting defects. When liquid supply is insufficient during solidification, the volumetric contraction of molten metal causes shrinkage porosity. Areas with poor cooling rates and minimal temperature gradients are especially vulnerable. Several studies use Niyama criteria, which connect the local temperature gradient to the cooling rate as a measure of shrinkage susceptibility, to quantify this behavior. Kim et al. used the Niyama criterion in ProCAST simulations to assess the likelihood of shrinkage in impeller castings, and they showed that areas with low Niyama values had a strong correlation with shrinkage problems that were discovered in experiments^1,6^.

Their findings demonstrated that, even when the riser size is greatly reduced, appropriate directional solidification toward the riser is necessary to remove shrinkage porosity. During mold filling, air entrainment and turbulent flow are the main causes of gas porosity and inclusion flaws. According to Jurkovicé et al., local velocity spikes brought on by abrupt changes in cross-sectional areas at the impeller inlet encouraged turbulence and oxide film entrainment^4^. It was demonstrated that these factors considerably raise the likelihood of defects in areas close to thick hubs and thin blades. Thermal stresses produced during restricted solidification are the cause of thermomechanical flaws such hot tearing and distortion. Wide solidification intervals, harsh mold conditions, and uneven cooling all contribute to stress accumulation, which is why thermomechanical analysis is crucial to defect prediction^10^.

Tools for casting simulation have been extremely useful for comprehending and improving intricate casting procedures. Numerical modeling of mold filling, heat transfer, solidification, and defect generation within a single framework is made possible by commercial software programs like ProCAST, MAGMASOFT, and Any Casting. Because of its finite element-based formulation and integration with thermodynamic databases, ProCAST is one of these that is frequently utilized in both academic and industrial research^5^.

To model mold filling, solidification behavior, and defect generation in investment-cast impellers, Kim et al. used ProCAST. The runner-riser system and mold tilt angle were redesigned with the help of their simulations, which accurately anticipated shrinkage, gas, and inclusion flaws. Ultrasonic testing was used to experimentally evaluate the numerical results, demonstrating the dependability of simulation-based process optimization^11^.

The location and degree of shrinkage porosity seen in actual castings were also faithfully replicated by numerical simulations when Jurković et al. used ProCAST to investigate sand-cast stainless-steel impellers. According to their study, simulation enables the assessment of several design options without the necessity for expensive physical testing, which significantly cuts down on development time and scrap rate^12^.

Modern casting modeling relies heavily on finite element analysis because it makes it possible to solve coupled governing equations for mass, energy, and momentum conservation accurately predicts hot spots and areas that are prone to shrinkage in solidification modeling by capturing feeding behavior, phase fraction evolution, and transient temperature fields. Typical simulations for stainless steel impellers include latent heat values of around 273 kJ/kg and temperature-dependent thermal conductivity values that range from about 26 W/m·K at pouring temperature to 14 W/m·K at room temperature^12^.

FEA is being used more in thermomechanical modeling in addition to thermal analysis to forecast residual stresses, deformation, and crack susceptibility offers crucial insight into defect mechanisms that thermal analysis alone is unable to capture by combining solidification shrinkage with mechanical constraints imposed by the mold. A significant step toward predictive, optimization-driven impeller casting is the integration of flow simulation, solidification modeling, and thermomechanical analysis inside a single FEA framework^12^.

This study specifically examines filling behavior, temperature distribution, cooling rate, solidification sequence, and shrinkage porosity prediction in impeller casting, despite previous research identifying hot tearing, deformation, thermal stress, and strain as significant casting-related defects. Consequently, the existing simulation framework was restricted to thermal-flow and solidification analysis, rather than encompassing a fully linked thermo-mechanical study. The discourse on stress-related flaws is presented solely as a general context to underscore the wider significance of casting simulation. A comprehensive assessment of thermal stress, strain, hot tearing propensity, and deformation will be conducted in further research utilizing integrated thermo-mechanical models and experimental verification.

The study aims to accomplish a series of cohesive objectives. A conservative baseline casting design (Design 1) is produced and subjected to numerical analysis to identify crucial sections susceptible to defects and inefficiencies in feeding and filling behavior. Secondly, a sequence of optimal designs (Designs 2–6) is methodically created by altering riser dimensions, gating geometry, mold tilting angle, and ventilation approach. The impact of these adjustments on temperature distribution, cooling rate, solidification sequence, and defect likelihood is quantitatively assessed by ProCAST simulations and defect prediction criteria. The assessment of casting yield and internal soundness across all designs aims to discover the ideal configuration that guarantees directional solidification, stable filling, and low material waste. The project seeks to establish that simulation-guided design iteration can supplant conventional trial-and-error methods for intricate impeller castings.

## Methodology and numerical method

### Overall computational workflow

To reduce casting flaws and increase material efficiency during the sand casting of a stainless-steel impeller, a systematic, simulation-driven workflow was used in this study. The process starts with creating a three-dimensional computer-aided design (CAD) model of the impeller and its related casting system, which includes the sprue, runner, gates, and risers. This first setup, known as Design 1, is a cautious baseline design that prioritizes fault prevention over material efficiency.

ProCAST was then used to predict mold filling, heat transfer, solidification behavior, and defect generation in finite element-based casting simulations. Quantitative defect indicators, including shrinkage porosity %, temperature distribution, cooling rate, and void location, were used to assess the outcomes of each simulation. A series of optimized designs, from Design 2 to Design 6, were produced as a result of specific changes made to the casting system geometry and process parameters in response to these findings. Until a casting with no flaws and increased material efficiency was attained, this iterative cycle of simulation, assessment, and redesign was maintained.

### Geometry and casting system description

The investigated component is a centrifugal pump impeller characterized by complex internal flow passages, variable wall thickness, and a central hub, which collectively pose challenges for uniform mold filling and directional solidification. The casting system was designed for gravity sand casting and comprised a pouring basin, vertical sprue, horizontal runner network, multiple ingates, and a feeding riser positioned above the hub region.

In the baseline configuration (Design 1), the casting system incorporated oversized gating elements and a large riser to ensure sufficient feeding during solidification. While this approach effectively eliminated shrinkage defects, it resulted in excessive material usage and prolonged solidification time. Across subsequent designs, geometric modifications were applied progressively to the riser dimensions, sprue geometry, runner layout, and gate positioning to improve flow stability and feeding efficiency while reducing non-functional metal volume. In later design stages, the central hole of the impeller was removed from the casting geometry and planned for post-casting machining, simplifying flow paths and enhancing solidification uniformity.

### Material models

The component under investigation is a centrifugal pump impeller, which presents difficulties for consistent mold filling and directed solidification due to its intricate internal flow passageways, variable wall thickness, and central hub. A pouring basin, vertical sprue, horizontal runner network, several ingates, and a feeding riser above the hub area made up the casting system, which was made for gravity sand casting.

To guarantee enough feeding during solidification, the casting system in the baseline configuration (Design 1) included a big riser and enlarged gating elements. Although this method successfully removed shrinkage problems, it used too much material and took a long time to solidify. To increase flow stability and feeding efficiency while lowering the volume of non-functional metal, geometric changes were gradually made to the riser size, sprue geometry, runner arrangement, and gate positioning in later iterations. Later design stages simplified flow routes and improved solidification uniformity by removing the impeller’s central hole from the casting geometry and planning for post-casting machining.

The casting material used in the numerical simulations was stainless steel CF8M, which is commonly used for corrosion-resistant pump and valve components. Since the prediction of solidification behavior and shrinkage porosity is strongly affected by the thermophysical properties of the alloy, the material model was defined using temperature-dependent properties where available in the ProCAST material database. The most relevant properties used in the simulation are summarized in Table 1.

The selected properties directly influence the calculated temperature field, cooling rate, solidification time, liquid feeding behavior, and shrinkage porosity tendency. In particular, the liquidus and solidus temperatures define the solidification interval, while the latent heat of fusion controls heat release during phase transformation. Thermal conductivity and specific heat affect the rate of heat extraction through the mold, whereas density and solidification shrinkage influence the feeding requirement during solidification.

The values in Table 1 denote the material input utilized to characterize the thermal and solidification behavior of the casting alloy. Temperature-dependent values were utilized where accessible in the simulation database, and indicative ranges are documented to enhance transparency. The same properties were systematically applied throughout all six examined gating and feeding designs, ensuring that the observed variations in shrinkage porosity, cooling rate, and solidification behavior could be ascribed to design alterations rather than fluctuations in material input.

In the simulation, the molten alloy was set at the designated pouring temperature, whilst the mould was set at ambient temperature. The alloy was regarded as an incompressible Newtonian fluid throughout the filling process and as a temperature-sensitive solidifying substance during cooling. The latent heat emitted between the liquidus and solidus temperatures was incorporated using the enthalpy-based solidification model, enabling the simulation to accurately represent the mushy zone and delineate final solidifying areas prone to shrinkage porosity.

**Table 1:** Thermophysical and solidification properties of stainless steel CF8M used in the ProCAST simulation.

Property	Symbol	Value used in simulation	Unit
Alloy	—	Stainless steel CF8M	—
Density	ρ	7700–7800	kg/m³
Liquidus temperature	T_L_	1450–1480	°C
Solidus temperature	T_S_	1370–1400	°C
Pouring temperature	T_p_	1650	°C
Initial mold temperature	T_m_	25	°C
Thermal conductivity	k	Temperature-dependent, approximately 15–30	W/m·K
Specific heat capacity	C_p_	Temperature-dependent, approximately 500–750	J/kg·K
Latent heat of fusion	L	approximately 270	kJ/kg
Dynamic viscosity	µ	Temperature-dependent	Pa·s
Solidification shrinkage	β_s_	Material-database value	%
Mold material	—	Silica sand	—

### Simulation environment, mesh strategy, solver settings, and boundary conditions

All numerical simulations were conducted using ProCAST, which utilizes the finite element approach to address coupled temperature, fluid flow, and mechanical issues. The computational domain encompassed the impeller, gating and riser systems, as well as the adjacent mold. A three-dimensional adaptable mesh was created, with refinement focused on crucial areas such as the blade-hub junctions, gating channels, and riser, where significant temperature gradients and intricate flow patterns were anticipated.

The numerical setup was organized into two parts: boundary and initial conditions, and solver-control settings. This separation was adopted to avoid confusion between physical boundary conditions and numerical time-stepping parameters.

#### Initial and boundary conditions

The computational domain consisted of the impeller casting cavity, sprue, runner, gates, riser/feeder, and surrounding silica sand mold. The molten stainless steel CF8M was initialized at the pouring temperature of 1650 °C, while the silica sand mold was initialized at 25 °C. Mold filling was modeled under gravity-driven conditions. A prescribed inlet velocity was applied at the sprue entrance to control the metal flow into the mold cavity. Heat transfer between the molten metal and the mold was represented using a casting–mold interfacial heat-transfer condition, while heat loss from the external mold surfaces was modeled using convective air-cooling boundary conditions.

#### Time-stepping and solver-control settings

The transient filling and solidification calculations were performed using automatic time integration in ProCAST as shown in Table 2. In this context, the maximum number of time steps refers to the maximum number of calculation increments allowed during the simulation, while the time-step size refers to the time increment advanced by the solver at each calculation step^13^.

The maximum number of time steps was set to 50,000 to provide sufficient numerical allowance for the filling, solidification, and cooling stages. The initial time-step size was set to 1 s, which defines the first-time increment used at the start of the calculation. The maximum time-step size was also limited to 1 s to prevent excessively large increments during the transient thermal calculation. Therefore, the value of 1 s refers to the time increment size, not to the number of time steps. The simulation was terminated according to the selected thermal stopping criterion after the casting reached the specified final temperature condition.

**Table 2:** Numerical time-stepping and solver-control settings used in the ProCAST simulation.

Parameter	Meaning	Value used
Maximum number of time steps	Maximum number of calculation increments allowed by the solver	50,000
Initial time-step size	First time increment used at the start of the transient calculation	1 s
Maximum time-step size	Upper limit imposed on the time increment during the calculation	1 s
Filling mode	Physical filling condition applied during mold filling	Gravity filling
Stopping criterion	Condition used to terminate the thermal calculation	Final temperature criterion
Evaluated stages	Simulation stages covered by the transient calculation	Filling, solidification, and cooling

The mold was set at 25 °C, Table 3 shows simulation parameters used in gravity filling analysis.Boundary conditions comprised a specified inflow velocity at the sprue entry, determined to attain a total filling duration of roughly 20 s, and convective heat dissipation from the external mold surfaces via film-coefficient-based air cooling. The alloy was characterized as linearly elastic to facilitate thermal stress assessment, but the mold was regarded as mechanically rigid.

The numerical simulation was conducted under gravity filling conditions using predefined time-stepping and stopping criteria to ensure numerical stability and accurate capture of the filling and solidification stages. Appropriate limits for the initial, filling, and maximum time steps were selected, while consistent units for temperature, heat flux, velocity, and pressure were defined to allow reliable post-processing and comparison of thermal–fluid results^14^.

**Table 3:** Simulation parameters used in gravity filling analysis.

Parameter	Description	Type	Value	Unit
NSTEP	Stop criterion: Maximum number of steps	Constant	50,000	—
TSTOP	Stop criterion: Final temperature	Constant	1.3090 × 10³	°C
DTI	Initial time step	Constant	1.0000 × 10⁻³	s
DTMAXFIL	Maximum time step during filling	Constant	1.0000 × 10⁻¹	s
DTMAX	Maximum global time step	Constant	1.0000 × 10⁰	s

The mold and casting volumes were specified together with their corresponding materials and beginning heat conditions to accurately simulate the investment casting environment as shown in Table 4. Boundary conditions were implemented to simulate air-cooling heat transfer at the exterior mold surfaces, a specified pouring temperature, and an inlet velocity condition to precisely predict heat dissipation, molten metal inflow, and thermo-mechanical behavior during the filling phase.

**Table 4:** Volume definition and boundary conditions used in the numerical simulation.

Category	Name	Type	Material/Entity	Fill %	Initial Temperature (°C)	Boundary Condition	Area (mm²)	Stress/Notes
Volume	Mold	Mold	Silica Sand	100.00	25.00	—	—	Rigid
Volume	Model	Alloy	Stainless Steel CF8M	0.00	1650.00	—	—	Linear Elastic
Boundary	Heat_1	Heat	EXT_Mold	—	—	Air cooling (film condition)	59,794.40	Heat loss to ambient
Boundary	Temperature_1	Temperature	USER_Temperature_1	—	1650.00	Prescribed temperature	530.99	Pouring temperature
Boundary	Velocity_1	Velocity	USER_Temperature_1	—	—	BC_Velocity_15	530.99	Inlet velocity condition

The inlet velocity was determined using the integrated velocity calculator, which relied on the designated filling time, inlet cross-sectional area, and the assumption of gravity-driven flow. This method guarantees a regulated and realistic filling rate of the molten metal, reducing excessive turbulence while ensuring complete mold filling within the designated timeframe. Pouring and filling parameters used in the numerical simulation were shown in Table 5.

**Table 5:** Pouring and filling parameters used in the numerical simulation.

Parameter	Description	Value	Unit
Computer Mode	Selected calculation mode	Fill Time	—
Velocity	Inlet molten metal velocity	8.77	mm/s
Area	Inlet cross-sectional area	530.99	mm²
Fill Limit	Target filling percentage	100	%
Flow Direction	Pouring direction	Gravity	—
Fill Time	Total mold filling time	20.0	s
Governing Relation	Filling time equation	Fill Time (s) = $\:\frac{Volume\:X\:Fill\:Limit\:\%}{100\:X\:Area\:X\:Velocity}$	s

Because shrinkage porosity prediction is sensitive to the resolution of the computational mesh, a mesh sensitivity analysis was performed to ensure that the numerical results were not strongly dependent on the selected element size. Three mesh densities were evaluated, namely coarse, medium, and fine meshes. The comparison focused on the predicted shrinkage porosity distribution, maximum shrinkage porosity percentage, temperature field, cooling-rate distribution, and location of the final solidifying regions.

The coarse mesh was used only for preliminary evaluation because it captured the general filling and solidification trends but provided less detailed resolution near the blade–hub junctions and feeder connection as explained in Table 6. The medium mesh improved the resolution in critical regions such as the hub, blade–hub junctions, gates, and riser neck. The fine mesh provided additional refinement in these regions but required a substantially higher computational cost.

The mesh sensitivity results showed that the predicted locations of shrinkage porosity and final solidification remained almost unchanged when the mesh was refined from the medium to the fine level. Only minor variations were observed in the maximum shrinkage porosity value and local cooling-rate magnitude. Therefore, the medium mesh was adopted for the full set of simulations because it provided a good compromise between accuracy and computational efficiency.

Based on this comparison, the medium mesh was considered sufficiently accurate for comparing the six gating and feeding configurations. Since the defect locations, final solidifying regions, and overall thermal trends did not change significantly between the medium and fine meshes, the conclusions regarding the improvement from Design 1 to Design 6 are not considered to be mesh-dependent.

**Table 6:** Mesh sensitivity analysis for shrinkage porosity prediction.

Mesh level	Relative mesh density	Main refinement regions	Predicted shrinkage porosity trend	Final solidification location	Assessment
Coarse mesh	Low	General casting and mold	Captures general trend but with limited local detail	Hub/feeder region	Used for preliminary screening
Medium mesh	Moderate	Hub, blade–hub junctions, gates, and riser neck	Stable porosity prediction with improved local resolution	Same as fine mesh	Selected for final simulations
Fine mesh	High	Further refinement of critical thermal-gradient regions	Only minor change compared with medium mesh	Same as medium mesh	Used to confirm mesh independence

Table 7 summarizes the complete numerical setup used in the ProCAST simulations for comparing the six impeller casting designs. The computational domain included the full casting system, consisting of the impeller, sprue, runner, gates, riser/feeder, and surrounding mold. Stainless steel CF8M was assigned as the casting alloy, while silica sand was used as the mold material. The molten alloy was initialized at a pouring temperature of 1650 °C, and the mold was initialized at 25 °C. Mold filling was modeled under gravity conditions, with a prescribed inlet velocity applied at the sprue entrance to achieve an approximate filling time of 20 s. Heat loss from the external mold surfaces was represented by convective air cooling, while thermal interaction between the casting and mold was defined using an interfacial heat-transfer condition. The mesh was refined in critical regions such as the hub, blade–hub junctions, gates, riser neck, and feeder region to improve the accuracy of thermal and defect predictions. The main simulation outputs included temperature distribution, cooling rate, solidification sequence, shrinkage porosity percentage, void distribution, and hot-spot locations, which were used to compare Designs 1–6 under consistent numerical conditions.

**Table 7:** Summary of model setup, initial conditions, boundary conditions, and numerical settings used in the ProCAST simulations.

Category	Parameter	Description/value used
Geometry	Computational domain	Impeller, sprue, runner, gates, riser/feeder, and surrounding mold
Casting alloy	Material	Stainless steel CF8M
Mold material	Material	Silica sand
Initial condition	Alloy temperature	Pouring temperature, 1650 °C
Initial condition	Mold temperature	25 °C
Filling condition	Filling mode	Gravity filling
Inlet boundary condition	Sprue inlet	Prescribed inlet velocity applied at the pouring inlet
Filling time	Total filling duration	Approximately 20 s
Thermal boundary condition	External mold surfaces	Convective air cooling
Thermal contact	Casting–mold interface	Interfacial heat-transfer condition
Mesh strategy	Refined regions	Hub, blade–hub junctions, gates, riser neck, and feeder region
Solver outputs	Thermal results	Temperature field, cooling rate, and solidification sequence
Defect outputs	Porosity prediction	Shrinkage porosity percentage, void distribution, and hot-spot locations
Design comparison	Evaluated cases	Six gating and feeding configurations, Design 1 to Design 6

This systematic setup was applied consistently to all six investigated designs. Therefore, the differences observed in temperature distribution, cooling behavior, solidification sequence, and shrinkage porosity formation can be attributed mainly to the changes in gating and feeding design rather than inconsistencies in the numerical setup.

**Fig. 1:**
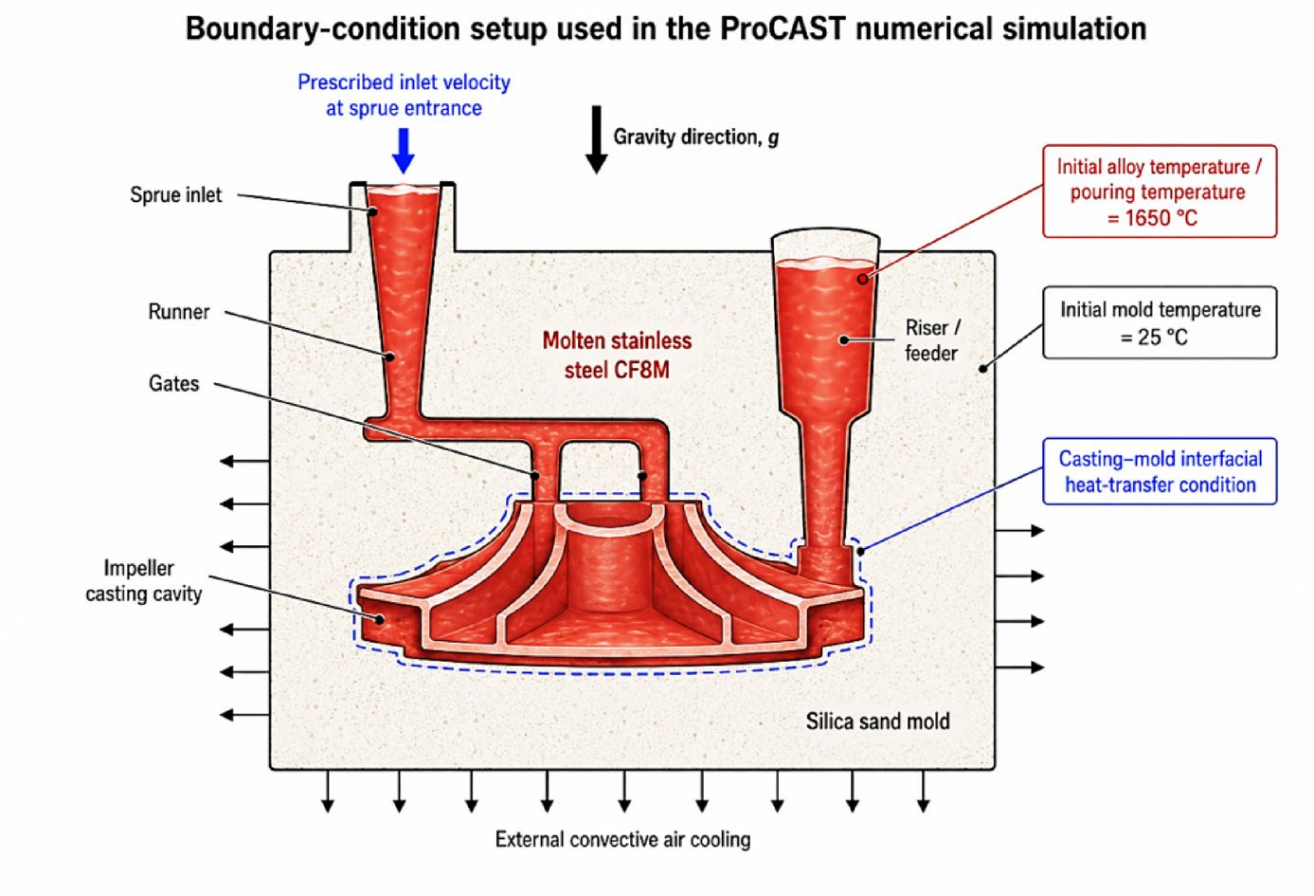
Schematic representation of the boundary-condition setup used in the ProCAST numerical simulation, showing the prescribed inlet velocity at the sprue entrance, gravity filling direction, initial alloy and mold temperatures, casting–mold interfacial heat-transfer condition, and external convective air-cooling boundary applied to the mold surfaces.

To improve the clarity of the numerical model setup, a schematic representation of the applied boundary conditions is provided in Fig. 1. The figure identifies the prescribed inlet velocity applied at the sprue entrance, the gravity-driven filling direction, the initial molten alloy temperature, the initial mold temperature, the casting–mold interfacial heat-transfer condition, and the convective air-cooling boundary condition applied to the external mold surfaces. This schematic was added to clarify how the computational domain was initialized and constrained during the filling, heat-transfer, and solidification stages of the ProCAST simulation.

Figure 1 illustrates the boundary-condition configuration employed in the ProCAST numerical simulation of the stainless-steel impeller casting. The computational domain encompasses the impeller casting chamber, sprue, runner, gates, riser/feeder, and the adjacent silica sand mould. The molten stainless steel CF8M was introduced via the sprue inlet, where a specified inlet velocity was utilized to regulate the mold-filling process. The filling process was simulated under gravity-driven conditions, as evidenced by the downward gravitational vector. The molten alloy’s initial temperature was established at 1650 °C, but the first mould temperature was designated as 25 °C. Heat transfer between the molten metal and the mould was depicted through a casting-mold interfacial heat-transfer condition, illustrated by the emphasized interface surrounding the casting cavity. Heat dissipation from the external mould surfaces was simulated via convective air-cooling boundary conditions, depicted by outward arrows around the mould. This configuration was uniformly implemented across all examined designs to guarantee that variations in filling behaviour, cooling rate, solidification sequence, and shrinkage porosity predictions were primarily ascribed to alterations in the gating and feeding systems, rather than modifications in numerical boundary conditions.

### Governing equations

The numerical model is governed by coupled conservation equations describing heat transfer, fluid flow, solidification, and thermo-mechanical behavior. Heat transfer within the casting and mold is governed by Fourier’s law of heat conduction Eq. (1):

1 $$\:\rho\:{c}_{p}\frac{\partial\:T}{\partial\:t}=\nabla\:\cdot\:(k\nabla\:T)+Q$$

where $\:\rho\:$is density, $\:{c}_{p}$is specific heat capacity, $\:T$is temperature, $\:k$is thermal conductivity, and $\:Q$represents latent heat release during solidification.

Molten metal flow during mold filling is described by the incompressible Navier–Stokes equations, ensuring conservation of mass and momentum. These equations account for gravity, pressure gradients, and viscous effects, enabling prediction of flow velocity, turbulence, and free-surface evolution.

Solidification is evaluated through phase-fraction-based models coupled with thermal analysis, allowing identification of hot spots and feeding-limited regions. Thermal stresses are computed using thermo-elastic formulations, where total strain is decomposed into elastic and thermal components arising from temperature gradients and constrained shrinkage during cooling.

#### Molten metal flow during filling

During mold filling, the molten stainless steel was assumed to behave as an incompressible Newtonian fluid. The conservation of mass is expressed as:

2 $$\:\nabla\:\:\cdot\:\mathrm{u}\hspace{0.17em}=\hspace{0.17em}0$$

where **u** is the molten metal velocity vector.

The momentum conservation equation is given by the incompressible Navier–Stokes equation:

3 $$\:\rho(\partial\:\mathrm{u}/\partial\:\mathrm{t}\hspace{0.17em}+\hspace{0.17em}\mathrm{u}\:\cdot\:\nabla\:\mathrm{u})\:=\:-\nabla\:\mathrm{p}\hspace{0.17em}+\mu\hspace{0.17em}\nabla^2\mathrm{u}\hspace{0.17em}+\hspace{0.17em}\rho\mathrm{g}$$

where p is the pressure, µ is the dynamic viscosity, and g is the gravitational acceleration vector.

These equations describe the filling behavior of molten metal inside the mold cavity and allow prediction of flow velocity, flow convergence, turbulence-prone regions, and possible gas or inclusion entrapment zones^15^.

#### Solidification and latent heat treatment

Solidification was modeled using a temperature-dependent solid fraction approach. During the liquid-to-solid transformation, latent heat release was included using the enthalpy formulation. The total enthalpy can be expressed as:

4 $$\:\mathrm{H}\left(\mathrm{T}\right)\:=\:\int\:\:\rho\mathrm{C}\mathrm{p}\left(\mathrm{T}\right)\mathrm{d}\mathrm{T}\hspace{0.17em}+\hspace{0.17em}\rho\mathrm{L}\mathrm{f}_\mathrm{s}\:\:$$

where H(T) is the temperature-dependent enthalpy, L is the latent heat of fusion, and **fs** is the local solid fraction.

The solid fraction varies between zero and one, as follows:

fs = 0 for fully liquid metal,

0 < fs < 1 for the mushy zone,

fs = 1 for fully solid metal.

Tracking the solid fraction allows identification of the liquid region, mushy zone, final solidifying zones, and isolated liquid pockets. These regions are important because they control feeding efficiency and shrinkage porosity formation.

#### Shrinkage porosity prediction

Shrinkage porosity formation is mainly associated with insufficient feeding during solidification. Regions that solidify last, have low temperature gradients, and experience poor feeding are more likely to develop shrinkage defects. In this study, shrinkage porosity prediction was interpreted using the local solidification behavior and a Niyama-type criterion:

5 $$\:\mathrm{N}\mathrm{y}\hspace{0.17em}=\hspace{0.17em}\mathrm{G}\:/\:\surd\mathrm{R}$$

where Ny is the Niyama criterion value, G is the local temperature gradient, and R is the local cooling rate.

Lower values of the Niyama criterion indicate a higher tendency for shrinkage porosity because they represent weak directional solidification and insufficient feeding. Therefore, the predicted shrinkage porosity distributions were used to compare the six gating and feeding designs and to determine whether the final solidifying regions remained inside the impeller body or were shifted toward the riser/feeder.

#### Main physical assumptions

The following physical assumptions were adopted in the simulations:

1. The molten stainless steel was treated as an incompressible Newtonian fluid during mold filling.
2. Mold filling was assumed to occur under gravity-driven conditions.
3. Heat transfer inside the casting and mold occurred mainly by conduction.
4. Heat loss from the external mold surfaces was modeled using convective air-cooling boundary conditions.
5. Thermophysical properties, including thermal conductivity, viscosity, and specific heat, were considered temperature-dependent where available.
6. Solidification was modeled using a phase-fraction approach with latent heat release included through the enthalpy formulation.
7. Shrinkage porosity was evaluated based on solidification sequence, temperature gradient, cooling rate, and feeding availability.
8. The mold was assumed to be mechanically rigid compared with the casting during the thermal analysis.

This formulation provides the mathematical and physical basis for the ProCAST simulations and supports the comparative evaluation of the six gating and feeding configurations in terms of filling stability, directional solidification, cooling behavior, and shrinkage porosity tendency.

### Optimization strategy from design 1 to design 6

This study utilizes a sequential, simulation-guided design change optimization technique. Design 1 functioned as a conservative benchmark, guaranteeing flawless casting with enlarged gating and risers. Designs 2 and 3 concentrated on minimizing riser volume and optimizing gating geometry while preserving satisfactory feeding performance.

Designs 4 implemented larger riser diameters and modifications to pouring temperature to improve directional solidification. Design 5 had a tapered sprue to enhance flow stability and minimize defects caused by turbulence, whereas In Designs 6, the central aperture was eliminated from the casting geometry, and the riser volume was further improved.

Throughout the design rounds, significant geometric alterations encompassed a systematic diminishment and resizing of risers, the incorporation of sprue tapering to enhance flow stability, adjustments to gating pathways to mitigate turbulence, and the simplification of the impeller geometry through the elimination of the center aperture. Every alteration was driven by simulation results indicating temperature gradients, cooling rates, and defect localization, guaranteeing that adjustments specifically targeted recognized problem processes instead of depending on empirical trial-and-error methods.

The Table 8 delineates the incremental advancement of the impeller casting design from Design 1 to Design 6, emphasizing the impact of systematic alterations in the runner–riser configuration, mold orientation, and venting strategy on filling dynamics, defect occurrence, and casting yield.

Design 1 denotes the basic configuration featuring a substantial riser (about 133 mm) and horizontal mold orientation. While shrinkage porosity was effectively eradicated with adequate feeding, the large riser led to a diminished casting yield (about 41.5%). Furthermore, the horizontal filling condition resulted in significant flow convergence and pronounced velocity oscillations, heightening the probability of gas and inclusion defects.

In Design 2, the riser diameter was diminished to around 66 mm, and the gate arrangement was refined. This alteration directed solidification while markedly enhancing material efficiency, raising the yield to around 65.1%. Nevertheless, the horizontal orientation of the mold resulted in ongoing moderate turbulence and flow convergence, so maintaining a residual risk of gas entrapment.

Designs 3, 4, and 5 incorporate incremental mold tilting of 5°, 10°, and 15°, respectively, while preserving the optimum feeding system from Design 2. As the tilt angle increases, the filling behavior stabilizes, and velocity fluctuations diminish. As a result, gas and inclusion faults diminish from moderate to minimal levels. However, the lack of specialized ventilation hinders the total removal of trapped gases, even at elevated tilt angles.

Design 6 integrates the enhanced runner–riser system with a 20° mold inclination and the incorporation of ventilators. This arrangement yields stable, near-laminar filling with minimal flow convergence and nearly uniform velocity profiles. Consequently, shrinkage porosity, gas entrapment, and inclusion flaws are entirely eradicated, while achieving a casting yield of around 65%. Consequently, Design 6 is recognized as the superior configuration, attaining the optimal equilibrium between casting quality and material efficiency.

**Fig. 2:**
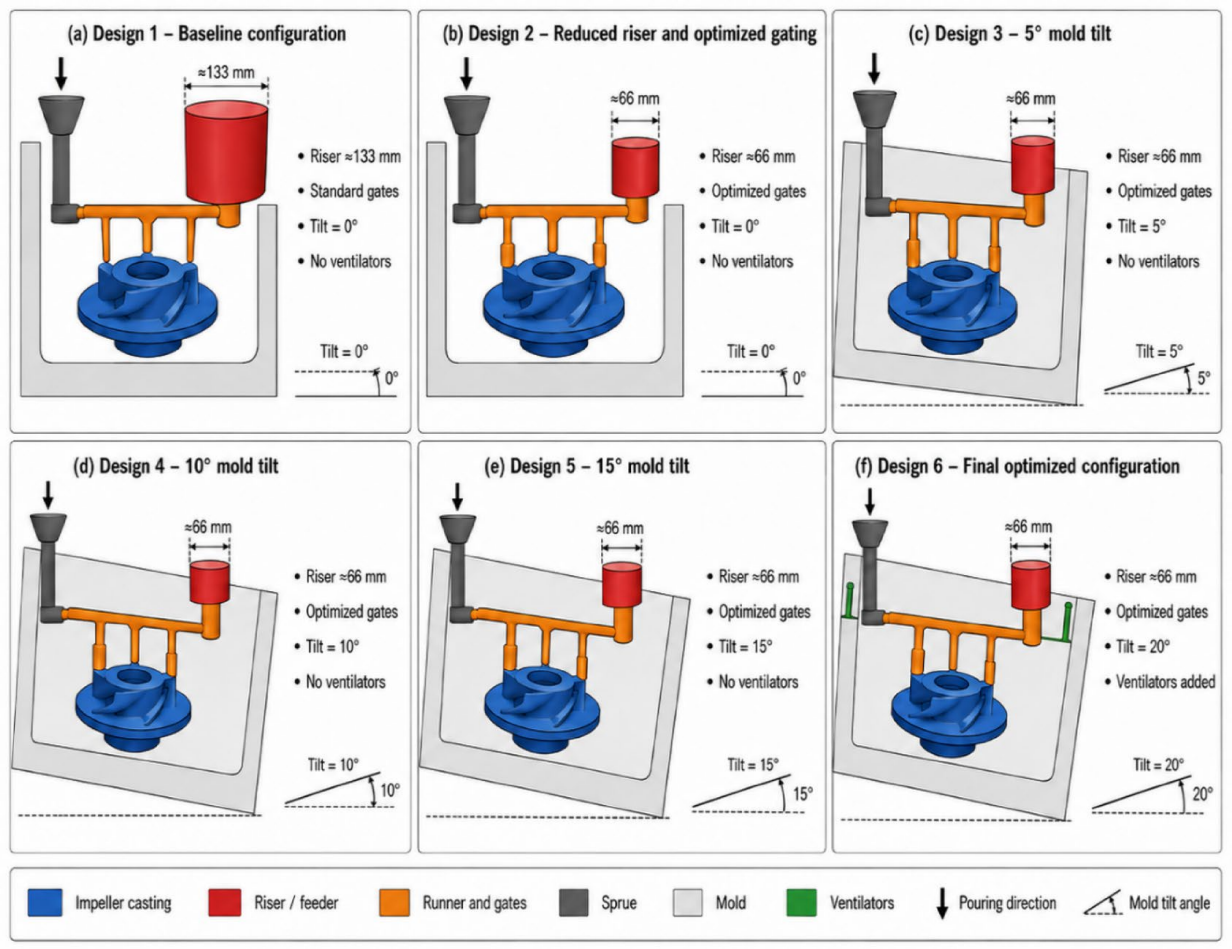
Schematic comparison of the six investigated gating and feeding system configurations for stainless-steel impeller casting.

To enable a clear comparison among the six examined casting configurations, schematic representations of Designs 1–6 have been shown in Fig. 2. The image succinctly illustrates the incremental alterations implemented in each instance, encompassing variations in riser diameter, gate configuration, runner design, mould tilt angle, incorporation of ventilators, and geometric simplification of the impeller. This graphical representation enhances Table 8 and elucidates the design progression from the conservative baseline setup to the final optimized casting method.

**Table 8:** Comparison of runner–riser configuration, mold tilting angle, filling behavior, defect tendency, and casting yield for impeller casting designs (Design 1–Design 6).

Parameter	Design 1	Design 2	Design 3	Design 4	Design 5	Design 6
Runner–riser concept	Conventional	Optimized	Optimized	Optimized	Optimized	Optimized
Riser diameter (mm)	≈ 133	≈ 66	≈ 66	≈ 66	≈ 66	≈ 66
Gate configuration	Standard size & location	Enlarged & elevated gates	Same as D2	Same as D2	Same as D2	Same as D2
Mold tilt angle (°)	0	0	5	10	15	20
Ventilators	None	None	None	None	None	Added
Filling behavior	Highly turbulent	Moderately turbulent	Improved stability	More uniform flow	Near laminar	Stable, near laminar
Flow convergence	Severe	Moderate	Reduced	Minor	Very minor	Eliminated
Velocity variation	High	Moderate	Reduced	Low	Very low	Minimal
Solidification pattern	Directional but inefficient	Directional	Directional	Directional	Directional	Fully directional
Shrinkage porosity	Eliminated	Eliminated	Eliminated	Eliminated	Eliminated	Eliminated
Gas & inclusion defects	High tendency	Moderate	Reduced	Low	Very low	Not observed
Casting yield (%)	≈ 41.5	≈ 65.1	≈ 64–65	≈ 64–65	≈ 64–65	≈ 65
Overall assessment	Safe feeding, poor yield	High yield, gas risk	Partial improvement	Better stability	Near optimal	**Optimal design**

### Defect evaluation criteria

Several quantitative indicators that were taken from ProCAST simulations were used to evaluate defects. The estimated volume fraction of shrinkage porosity, reported as a percentage, was used to evaluate shrinkage flaws. Using maps of temperature and solidification time, hot spots were found, emphasizing areas of delayed solidification. Directional solidification’s efficacy was assessed by analyzing cooling rate distributions.

**Fig. 3:**
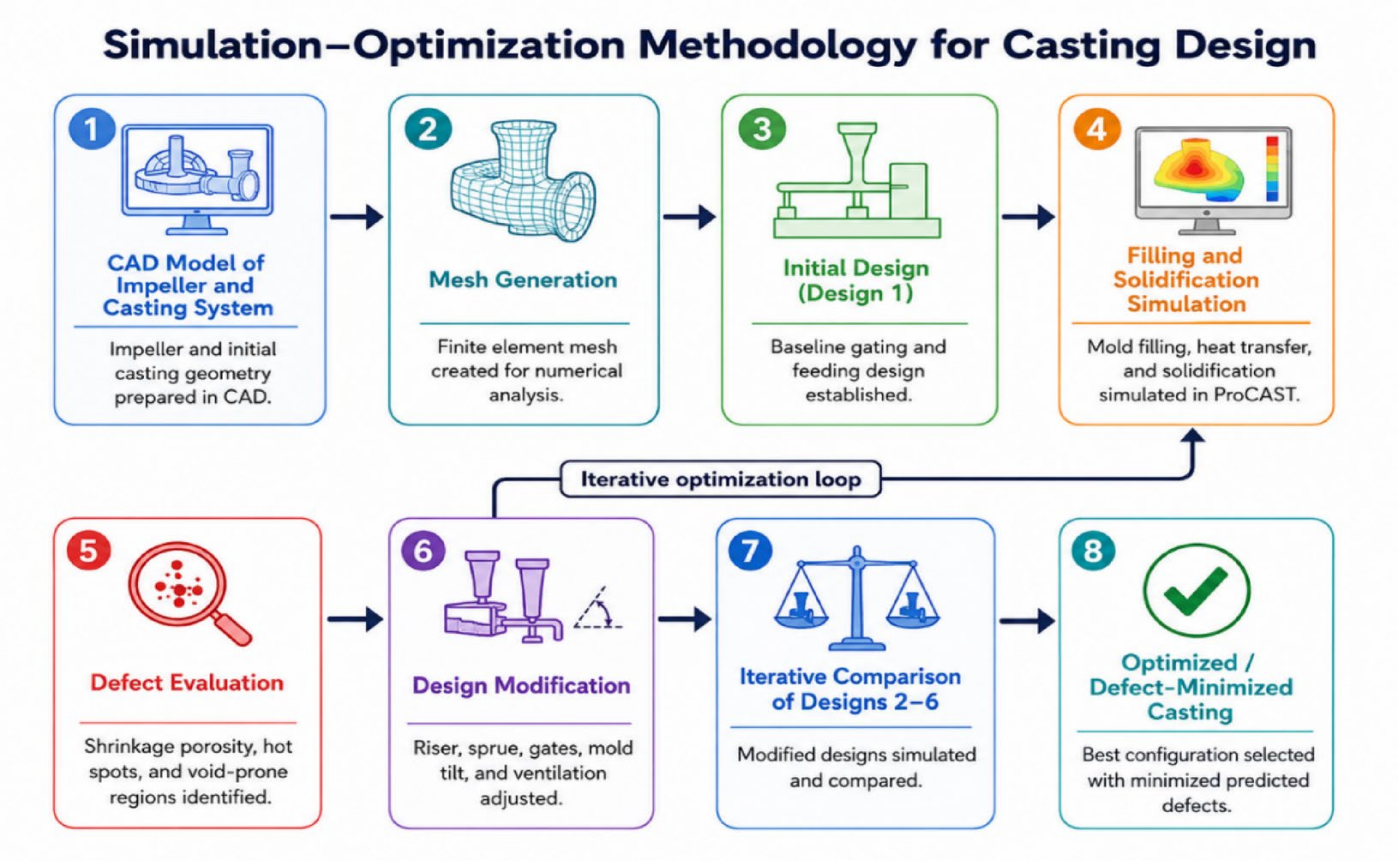
Simulation–optimization workflow for impeller casting design, showing CAD preparation, mesh generation, initial design simulation, defect evaluation, iterative design modification, comparison of Designs, and selection of the optimized defect-minimized casting configuration.

To differentiate between gas-related voids and shrinkage-related porosity, void occurrence and spatial distribution were investigated. When evaluating the quality of the casting, the position of flaws in relation to the impeller’s functional areas such as the hub and blade junctions were thought to be crucial. The selection of the final optimum configuration was guided by the objective comparison of all design iterations made possible by the combined application of these criteria. Figure 3 illustrates the sequential methodology integrating CAD modeling, finite-element simulation, defect evaluation, and iterative design refinement to achieve a defect-free casting.

## Results and discussions

### Shrinkage porosity evolution with gating and feeding design optimization

**Fig. 4:**
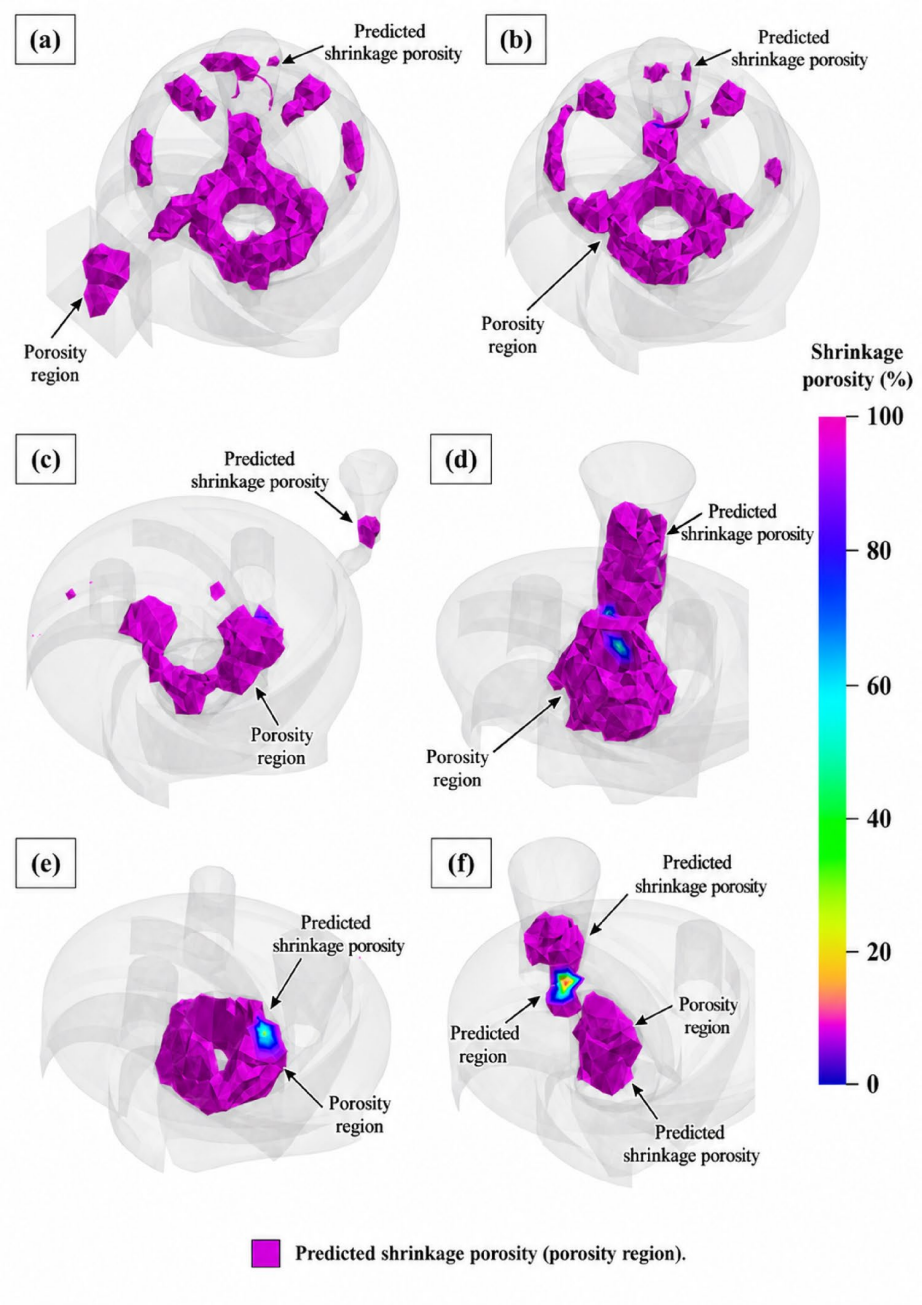
Predicted shrinkage porosity distributions for six different impeller gating and feeding designs obtained from ProCAST simulations: **(a)** Design 1, **(b)** Design 2, **(c)** Design 3, **(d)** Design 4, **(e)** Design 5, and **(f)** Design 6. The colored iso-surfaces represent regions with a high probability of shrinkage porosity, highlighting the influence of feeding efficiency and directional solidification on defect formation.

The shrinkage porosity distributions illustrated in Fig. 4(a–f) demonstrate a distinct advancement in defect reduction as the gating and feeding design is refined. Design 1 (a) exhibits significant and widespread shrinkage porosity in the hub and blade–hub connections, signifying inadequate feeding efficiency and a lack of directed solidification. The riser and gate layout in this design does not maintain an optimal thermal gradient, leading to several isolated hot spots and extensive volumetric shrinkage. A comparable phenomenon is observed in Design 2 (b), where shrinkage porosity is extensively dispersed, but somewhat more concentrated in the hub region. This indicates very slight enhancement in feeding efficiency, with solidification still taking place unevenly throughout the casting^16^.

A significant decrease in shrinkage porosity is observed in Design 3 (c), where flaws are more localized and predominantly restricted to the hub region. This signifies the partial implementation of directed solidification; nonetheless, the existence of isolated porosity regions implies that the feeding pathways are obstructed prior to the complete solidification of thicker parts. Design 4 (d) demonstrates enhanced performance, exhibiting shrinking porosity that creates an elongated area oriented along the feeding direction. This behavior indicates improved heat regulation and riser efficiency; however, the ongoing presence of porosity suggests that the feeder volume or solidification duration is inadequate to entirely offset shrinkage in the ultimate hot spot.

In Design 5 (e), shrinkage porosity is markedly reduced and confined to a limited area near the feeder, signifying nearly perfect feeding conditions and efficient directed solidification across most of the impeller. Only negligible residual porosity persists, presumably resulting from a tiny discrepancy between the riser modulus and the final solidifying zone. The optimal performance is attained in Design 6 (f), wherein shrinkage porosity is diminished to minimal, isolated areas predominantly situated inside the feeder zone. This verifies the effective implementation of directional solidification from the impeller body to the riser, guaranteeing sufficient feeding during the concluding phases of solidification and yielding a robust casting with minimum internal flaws^2^.

The identified shrinkage porosity patterns are directly correlated with the geometric and feeding attributes of each design. In Design 1, the traditional gating configuration and horizontal mould orientation yielded inadequate directional feeding towards the hub and blade-hub connections, leading to localized hot spots and extensive shrinkage porosity throughout the impeller body. Despite Design 2 using a diminished riser and an enhanced gate configuration, the horizontal filling situation continued to permit localized heat stagnation and inadequate feeding in thicker parts, resulting in residual porosity in the hub region. In Design 3, the implementation of a 5° mould tilt enhanced the filling trajectory and diminished flow convergence, hence localizing shrinkage porosity to fewer crucial areas. Elevating the mould tilt to 10° in Design 4 enhanced the temperature gradient between the impeller and feeder, facilitating more efficient directed solidification and relocating the final solidifying zone nearer to the riser. In Design 5, the 15° inclination resulted in enhanced filling stability and improved feeding continuity, hence confining shrinkage porosity to a localized area adjacent to the feeder. Ultimately, Design 6 integrated the optimized runner–riser system, a 20° mould tilt, and ventilators positioned at the higher gas-escape zones of the mould cavity. This arrangement offered the optimal feeding channel and heat gradient, facilitating the movement of the final solidifying region toward the riser/feeder and reducing shrinkage porosity within the functional impeller body. The gradual decrease in shrinkage porosity from Design 1 to Design 6 is ascribed to the synergistic effects of optimized gate configuration, increased feeding efficiency, regulated mould tilt, and improved expulsion of entrapped gases. To support the visual comparison of shrinkage porosity iso-surfaces, the predicted shrinkage porosity values were also quantified for each design, as summarized in Table 9.

**Table 9:** Quantitative comparison of predicted shrinkage porosity for the six investigated designs.

Design	Main design feature	Main porosity location	Defect tendency
Design 1	Baseline gating, large riser, 0° tilt	Hub/impeller body	High
Design 2	Reduced riser and optimized gates, 0° tilt	Hub region/feeder neck	Moderate to high
Design 3	Optimized gates with 5° mold tilt	Localized near hub/riser connection	Moderate
Design 4	Optimized gates with 10° mold tilt	Near feeder region	Low to moderate
Design 5	Optimized gates with 15° mold tilt	Mainly close to riser/feeder	Low
Design 6	20° mold tilt with ventilators	Mostly shifted to feeder/riser, minimal inside impeller	Lowest

### Effect of gating and feeding design on cooling rate distribution

**Fig. 5:**
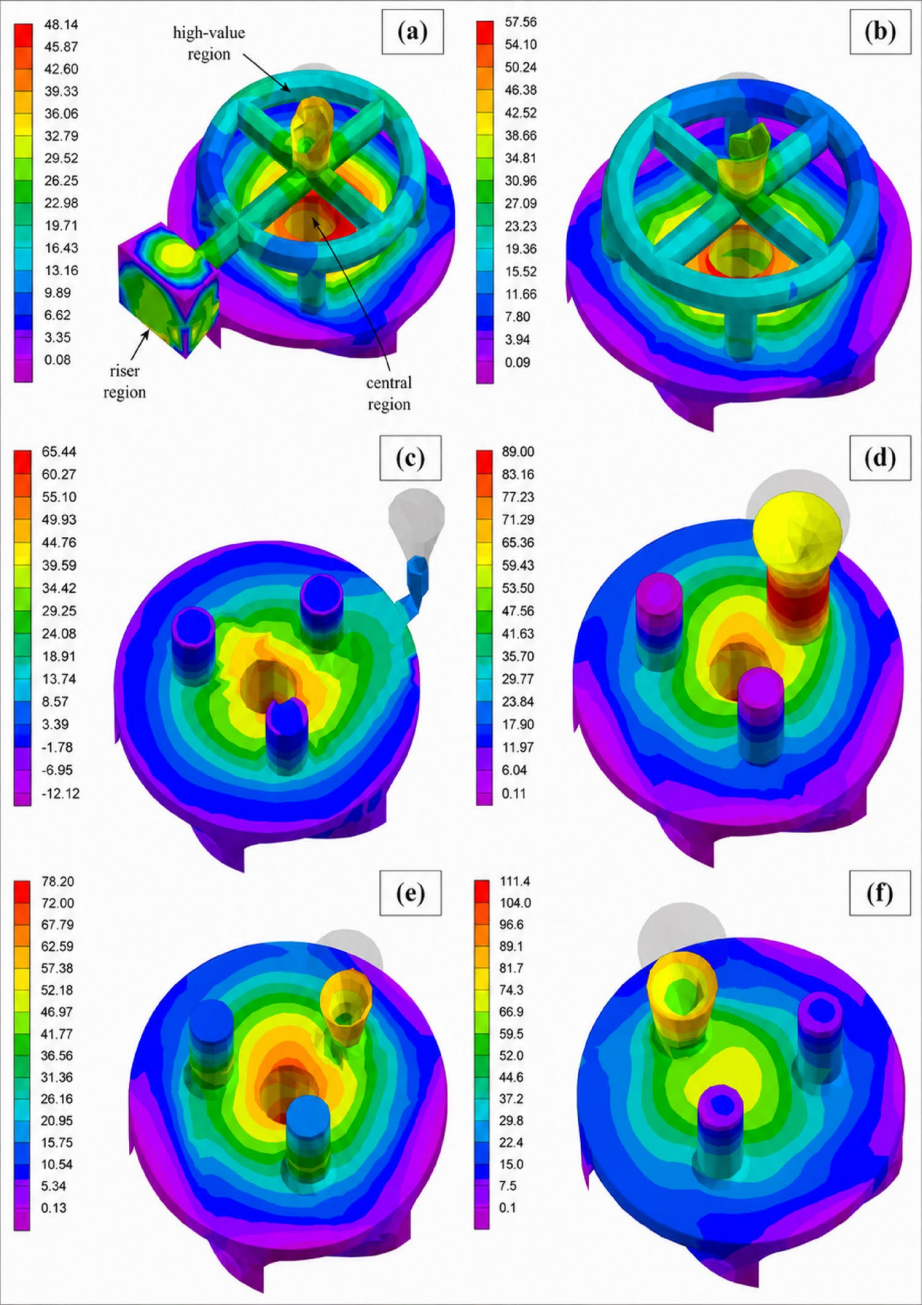
Cooling rate distributions of the impeller predicted by ProCAST for different gating and feeding configurations: **(a)** Design 1, **(b)** Design 2, **(c)** Design 3, **(d)** Design 4, **(e)** Design 5, and **(f)** Design 6. The color contours represent the local cooling rate, illustrating the evolution of thermal gradients and solidification behavior as the design is optimized.

The cooling rate distributions depicted in Fig. 5(a–f) offer critical insights into the solidification behavior of the impeller across the six examined designs and elucidate the observed trends in shrinking porosity. In Design 1 (a), the cooling rate exhibits considerable non-uniformity, characterized by elevated cooling rates in the outer thin portions and markedly reduced cooling rates in the hub and blade-hub junctions. The significant heat gradient fosters the development of distinct hot spots and prolongs solidification in the center areas, heightening the vulnerability to shrinkage flaws. The same pattern is evident in Design 2 (b), where the overall cooling rate distribution is inconsistent; despite some redistribution of thermal gradients, the hub region solidifies last, signifying inadequate regulation of heat extraction and feeding efficiency.

In Design 3 (c), the cooling rate distribution is more uniform in comparison to Designs 1 and 2. The center hub region continues to display diminished cooling rates compared to adjacent places, however the gradient between the hub and peripheral parts has lessened. This shows a partial enhancement in heat dissipation and solidification sequencing; nonetheless, the presence of a low cooling rate zone at the center signifies that final solidification transpires within the casting rather than the feeder. Design 4 (d) illustrates a more pronounced shift in solidification control, with elevated cooling rates prevalent over most of the impeller body, whilst the minimal cooling rates are localized around the feeder region. This behavior indicates a more advantageous thermal gradient that promotes directional solidification towards the feeder; however, the extent of the low cooling rate zone implies that feeding is not yet entirely optimized.

Design 5 (e) demonstrates enhanced uniformity in the cooling rate distribution over the impeller, with the lowest cooling rates localized to a reduced area near the feeder. This suggests that most of the impeller solidifies swiftly and uniformly, hence diminishing the probability of isolated hot patches inside the operational areas. Design 6 (f) attains the most optimum condition, demonstrating a smooth and well-regulated cooling rate gradient from the impeller body to the feeder. The primary casting areas exhibit moderate to high cooling rates, whilst the lowest cooling rates are confined to the feeder itself. This verifies the implementation of efficient directed solidification, guaranteeing that the feeder remains in a molten state for an extended duration and can sufficiently address volumetric shrinkage, thus reducing the likelihood of internal flaws^17^.

### Effect of gating and feeding design on temperature distribution and hot-spot formation

**Fig. 6:**
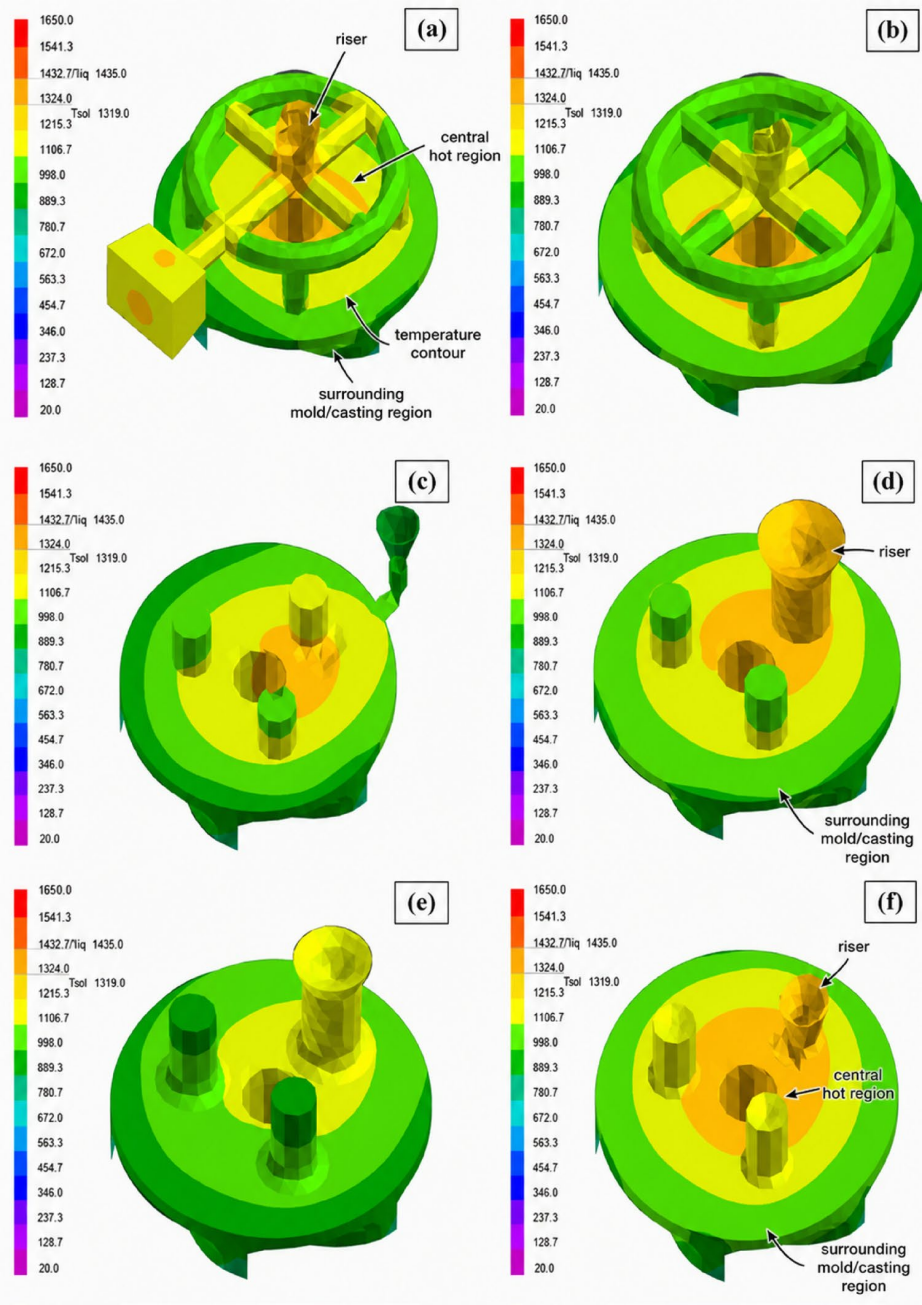
Temperature distribution during solidification of the impeller predicted by ProCAST for different gating and feeding designs: **(a)** Design 1, **(b)** Design 2, **(c)** Design 3, **(d)** Design 4, **(e)** Design 5, and **(f)** Design 6. The color contours represent the local temperature (°C), highlighting the location of hot spots and the evolution of thermal gradients as the design is optimized.

The temperature distribution results depicted in Fig. 6(a–f) demonstrate the progression of thermal fields throughout solidification for the six examined designs, elucidating the enhancements in feeding and defect management. In Design 1 (a), the temperature distribution is markedly heterogeneous, with maximum temperatures of ≈ 1450 °C concentrated at the hub and blade-hub junctions, whilst the peripheral portions cool to around 900–1000 °C. The significant temperature disparity signifies intense thermal gradients and the emergence of several hot spots inside the casting, which are susceptible to delayed solidification and shrinkage porosity. A comparable phenomenon is noted in Design 2 (b), wherein the center hub region sustains excessive temperatures of approximately 1400–1430 °C, but the adjacent areas fall below 1000 °C, indicating that the design alterations are inadequate to relocate the last solidifying zones towards the feeder^18^.

In Design 3 (c), the temperature distribution is more uniform, with the core hot spot temperature decreased to roughly 1350–1380 °C, while most of the impeller body stays within the range of 1000–1150 °C. The decrease in peak temperature and the more gradual gradient suggest a partial enhancement in heat extraction; still, the maximum temperatures remain within the casting instead of the feeder, indicating inadequate directed solidification. Design 4 (d) demonstrates a notable enhancement, with peak temperatures of approximately 1400–1450 °C relocated nearer to the feeder region, while the impeller body exhibits a more uniform cooling to around 1000–1100 °C. This thermal pattern indicates a more advantageous solidification sequence, albeit a considerable hot patch remains along the feeder–casting contact.

In Design 5 (e), the temperature distribution exhibits a more distinct delineation between the casting and the feeder. The impeller body maintains temperatures of roughly 950–1050 °C, however the feeder sustains higher temperatures between 1350 and 1450 °C, suggesting that the feeder remains molten considerably longer than the casting. This condition significantly facilitates directed solidification and diminishes the probability of interior hot spots. Design 6 (f) exhibits the most optimal thermal behavior, with peak temperatures over 1400 °C predominantly contained within the feeder, while the impeller body maintains a uniform cooling to around 900–1000 °C. The uniform thermal gradient from the casting to the feeder verifies efficient heat flow management, ensuring that the feeder solidifies last, which allows for sufficient compensation of volumetric shrinkage and reduces the likelihood of shrinkage-induced flaws^19^.

### Defect prediction analysis based on filling and solidification simulations

Defection prediction analysis was conducted using ProCAST to assess the probability of shrinkage porosity, gas entrapment, and non-metallic inclusions in the six examined designs. The investigation focused on the integrated evaluation of filling behavior, temperature distribution, cooling rate, and solidification features, specifically highlighting the Niyama criterion and flow stability indicators^20^.

The defect prediction results for Design 1 demonstrated a minimal likelihood of shrinking porosity owing to the inclusion of a substantial riser, which facilitated adequate feeding during solidification. The filling simulation demonstrated significant turbulence and intense flow convergence adjacent to the impeller cover and in the thin-walled areas. These circumstances are recognized to facilitate air entrainment and oxide formation, resulting in significant projected propensity for gas and inclusion defects, notwithstanding the lack of shrinkage-related concerns.

In Design 2, the optimized riser dimensions and gate arrangement preserved directed solidification and substantially diminished the possibility of shrinkage porosity, as evidenced by enhanced Niyama criteria values in crucial areas. However, due to the mold’s horizontal orientation, flow convergence zones continued to exist during the filling process. The anticipated probability of gas-related faults was diminished in comparison to Design 1 yet not entirely eradicated.

The use of mold tilting in Designs 3, 4, and 5 indicated a distinct trend of enhanced filling stability in the defect prediction study. Raising the tilt angle from 5° to 15° systematically diminished velocity gradients and flow impingement at geometric discontinuities. This resulted in a significant reduction in anticipated gas entrapment and inclusion development. Shrinkage porosity was nonexistent in all inclined configurations owing to the maintained optimum feeding mechanism. Nevertheless, localized areas with diminished venting performance nevertheless demonstrated slight gas retention, especially within enclosed impeller chambers^21^.

Design 6 demonstrated the most advantageous fault prediction outcomes. The integration of an optimized runner–riser system, a 20° mold inclination, and supplementary ventilators produced smooth, nearly laminar filling with negligible flow separation. The temperature gradients and cooling rates were effectively balanced, guaranteeing complete directional solidification towards the riser. Thus, the Niyama criterion values consistently above the critical threshold over the impeller volume, signifying minimal risk of shrinking porosity. The consistent filling pattern and efficient gas evacuation routes precluded the anticipated emergence of gas and inclusion faults.

### Limitations and future work

The current study provides a systematic numerical comparison of six gating and feeding configurations for stainless-steel impeller casting; however, direct experimental validation has not been performed at this point of the research. Consequently, the results have to be regarded as simulation-derived forecasts that highlight comparative design trends rather than as well proven experimental findings.

The lack of experimental validation constitutes a drawback, as the prediction of shrinkage porosity may be influenced by errors in material parameters, mould conditions, heat transfer coefficients, pouring techniques, mesh resolution, and boundary conditions. To overcome this restriction, subsequent efforts will entail fabricating the optimized casting design and juxtaposing the predicted defect predictions with empirical data.

The validation plan will incorporate non-destructive testing techniques, including X-ray radiography and ultrasonic examination, to identify internal shrinkage porosity and gas-related problems. Furthermore, chosen castings will be dissected at pivotal sites, specifically the hub, blade-hub junctions, feeder neck, and final solidification areas, to juxtapose the anticipated porosity locations with actual internal defects. Metallographic analysis and dimensional assessment will be conducted to determine casting integrity and geometric precision.

This forthcoming validation will furnish quantitative evidence regarding the predictive efficacy of the ProCAST model and will ascertain whether the optimized Design 6 can consistently mitigate shrinkage porosity and direct the final solidifying regions towards the feeder/venting system under practical casting conditions.

## Conclusions

- Six impeller casting designs were meticulously evaluated in this study by incrementally modifying the mould tilting technique and runner–riser configuration to optimize casting yield while minimizing shrinkage porosity, gas entrapment, and inclusion defects.
- Design 1 (baseline arrangement) included a substantial riser (about 133 mm in diameter) with a horizontal mould orientation. Despite the successful prevention of shrinkage porosity through adequate feeding, the substantial riser volume resulted in a low casting yield of around 41.5%, and the filling behavior demonstrated significant turbulence, which facilitated gas and inclusion defects.
- Design 2 optimized the feeding system by reducing the riser diameter to around 66 mm and altering the gate locations and dimensions. This configuration had the highest yield among non-tilted examples (~ 65.1%), representing an increase of approximately 23.6% points over Design 1, while ensuring defect-free solidification and significantly enhancing material efficiency. The potential for gas entrapment persisted, however, owing to flow convergence at the impeller cover.
- The optimized runner-riser system from Design 2 was preserved in Designs 3, 4, and 5, which incorporated mould tilting at angles of 5°, 10°, and 15°, respectively. Progressive tilting mitigated velocity variations and reduced flow instability relative to the horizontal configuration. Nonetheless, isolated turbulence and residual gas pockets were seen at tilt angles 15°, particularly near regions of sudden cross-sectional variation. Shrinkage porosity was well managed; nevertheless, gas-related defects were not entirely eliminated.
- Design 6, which integrates the modified runner–riser system, a 20° mould inclination, and additional ventilators, yielded the most optimal performance. The molten metal exhibited consistent, laminar filling characterized by nearly uniform velocity profiles and minimal variation in cross-sectional area throughout the filling height. This design effectively eliminated shrinkage porosity, gas entrapment, and non-metallic impurities while maintaining a yield comparable to Design 2 (about 65%). In comparison to Design 1, Design 6 exhibited superior internal soundness and a yield enhancement above 50%.
- The numerical simulations demonstrated that the gating and feeding design substantially influenced the temperature distribution, cooling behavior, and movement of the solidification front in the stainless-steel impeller casting. The initial designs concentrated the final solidification zones within the impeller structure, particularly around the hub and blade-hub connections, hence increasing the likelihood of isolated liquid pockets and shrinkage porosity.
- By methodically modifying the riser, sprue, gate layout, mould tilt angle, and ventilation system, the temperature gradient was enhanced, leading to a steady shift of the final solidification zone towards the riser/feeder region. The optimized design permitted directed solidification, improved feeding efficiency, reduced hot-spot accumulation within the operational impeller body, and minimized expected shrinkage porosity.
- This study demonstrates that virtual comparisons of different casting configurations can effectively replace iterative trial-and-error casting experiments, acting as a predictive tool for identifying defect-prone areas, guiding design modifications, and improving the casting process for stainless-steel pump impellers via numerical simulation.

## Data Availability

The datasets used and/or analyzed during the current study are available from the corresponding author on reasonable request.

## References

[1] Wang, C. et al. Influence of Impeller Structure Parameters on the Hydraulic Performance and Casting Molding of Spiral Centrifugal Pumps. *Water* **16** (11), 1598 (2024).

[2] Jurković, K. et al. Numerical simulation of sand casting of stainless steel pump impeller. *Metals* **14** (4), 435 (2024).

[3] Veera Raghavulu, K. et al. Residual stresses and microstructure analysis of SS316L impeller part using selective laser melting: simulation and experimental validation. *Int. J. Interact. Des. Manuf. (IJIDeM)*, **20**, 1845–1856 (2026).

[4] Iyer, S., Deshmukh, S. M. & Tapre, R. W. Enhancing impeller design parameters for optimal pump efficiency and performance in supercritical thermal power projects. *Energy Technol.*10.1002/ente.202500022 (2025).

[5] Mugeri, H. Optimisation of casting process of sand cast austenitic stainless-steel pump impeller using numerical modelling and additive manufacturing, 2018, Vaal University of Technology (South Africa).

[6] Akindapo, J., Shehu, G. & Orueri, D. Development of Aluminium Metal Matrix Composite for Production of Centrifugal Pump Impeller. *Acad. J. Sci. Eng.* **17** (2), 1–20 (2023).

[7] Kim, Y. C. et al. Improvement of quality and yield for investment casting of centrifugal pump impeller by tilting mold and optimizing runner/riser system. *Int. J. Adv. Manuf. Technol.* **130** (5), 2369–2379 (2024).

[8] Khan, M. A., Sheikh, A. K. & Asad, M. Mold design and casting of an impeller using MAGMASoft. *Int. J. Mech. Eng. Robot Res.* **9** (12), 1579–1583 (2020).

[9] Abdullin, A. Computer modeling of a large casting in the software package ProCAST. *Metallurgist* **56** (9), 721–726 (2013).

[10] Condruz, M. et al. Solidification simulation and casting of an impeller designed for a thermochemical treatment furnace. in AIP Conference Proceedings. AIP Publishing LLC. (2020).

[11] Condruz, M. et al. Computational and experimental study on investment cast micro turbo-pump impeller. in AIP Conference Proceedings. AIP Publishing LLC. (2022).

[12] Hernández, F. & Fragoso, A. Fabrication of a stainless-steel pump impeller by integrated 3D sand printing and casting: Mechanical characterization and performance study in a chemical plant. *Appl. Sci.* **12**(7), 3539 (2022).

[13] Zhang, L. et al. Ultrafine grain control by ultrasonic vibrations in directed energy deposition. *Int. J. Mech. Sci.*10.1016/j.ijmecsci.2025.110925 (2025).

[14] Zhang, L. & Zhang, Z. Particle motion control in ultrasonic assisted directed energy deposition. *Int. J. Mech. Sci.* **294**, 110259 (2025).

[15] Zhang, L. et al. Gas-particle-heat dynamic coupling simulation in directed energy deposition. *Int. J. Mech. Sci.* **275**, 109302 (2024).

[16] Tan, M. et al. Investigation on performance of a centrifugal pump with multi-malfunction. *J. Low Freq. Noise Vib. Act. Control* **40**(2), 740–752 (2021).

[17] Huang, P.-H. & Guo, M.-J. A study on the investment casting of 17-4PH stainless steel helical impeller of centrifugal pump. *Mater. Res. Innov.* **19**(sup9), S9-77-S9-81 (2015).

[18] Paladino, E. E. et al. Theoretical and experimental analysis of multiphase twin-screw pumps operating in serial arrangement. *J. Pet. Sci. Eng.* **216**, 110701 (2022).

[19] Jian, Z. & Chunlei, Y. Optimization of investment casting process for stainless steel impeller with complicated geometry. *Chin. J. Mater. Res.* **29** (12), 955–960 (2016).

[20] Nenchev, B. *Modelling and analysis of solidification shrinkage and defect prediction in metals* (University of Leicester, 2023).

[21] Chen, Z. et al. Progress in numerical simulation of casting process. *Meas. Control*. **55** (5–6), 257–264 (2022).

